# Right Ventricular Adaptation Assessed Using Cardiac Magnetic Resonance Predicts Survival in Pulmonary Arterial Hypertension

**DOI:** 10.1016/j.jcmg.2020.10.008

**Published:** 2021-06

**Authors:** Ze Ming Goh, Samer Alabed, Yousef Shahin, Alexander M.K. Rothman, Pankaj Garg, Allan Lawrie, David Capener, A.A. Roger Thompson, Faisal A.A. Alandejani, Christopher S. Johns, Robert A. Lewis, Krit Dwivedi, James M. Wild, Robin Condliffe, David G. Kiely, Andrew J. Swift

Pulmonary arterial hypertension (PAH) is a rare but life-limiting condition. Assessments of disease severity and prognosis are essential in the selection of treatment options and the timing of lung transplantation (1). Right ventricular (RV) volume and mass parameters measured using cardiac magnetic resonance have been suggested to be prognostic in PAH (1, 2, 3).

This study aimed to determine the prognosis of patients with PAH based on patterns of RV adaption in PAH using RV volume and mass. Ethical approval by the North Sheffield ethics committee and review board approval was obtained (reference c06/Q2308/8).

Consecutive patients who underwent cardiac magnetic resonance and were diagnosed with PAH from the ASPIRE (Assessing the Spectrum of Pulmonary Hypertension Identified at a Referral Centre) Registry between May 12, 2009 to February 1, 2015 were included. The patients were followed up until census or death. RV end-systolic volume was measured on short-axis cine images, corrected for body surface area, adjusted for sex and age, and presented as: RV end-systolic volume index percentage predicted (RVESVI_%pred_) (4). Ventricular mass index (VMI) was calculated as RV end-diastolic mass divided by left ventricular end-diastolic mass. Univariate Cox regression analysis was used to assess the prognostic value of RVESVI_%pred_ and VMI.

The patients were divided into 4 different volume/mass groups using a RVESVI_%pred_ threshold of 227% (1) and a median value of VMI because there was no known well-defined PAH prognostic threshold for VMI. The groups were as follows: Vol_low_Mass_low_ (low RVESVI_%pred_ and low VMI), Vol_low_Mass_high_ (low RVESVI_%pred_ and high VMI), Vol_high_Mass_low_ (high RVESVI_%pred_ and low VMI), and Vol_high_Mass_high_ (high RVESVI_%pred_ and high VMI). Kaplan-Meier plots, Cox regression analysis, and one-way analysis of variance were used to compare the prognoses and variables of different groups. Multivariate Cox regression analysis was used to identify prognostic indicators that were independent of volume/mass group. Subgroup analyses were performed in incident patients.

A total of 564 patients with PAH were identified; 250 (44%) died at follow-up (mean 5.0 ± 2.6 years). RVESVI_%pred_ (scaled hazard ratio [HR]: 1.323; 95% confidence interval [CI]: 1.183 to 1.480; p < 0.001) and VMI (scaled HR: 0.864; 95% CI: 0.757 to 0.985; p = 0.029) had prognostic value.

**Figure 1:**
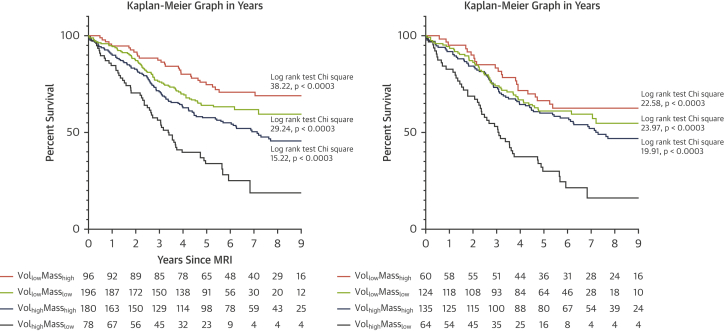
Kaplan-Meier Graphs Illustrating Survival of Patients by Volume/Mass Group in the Full and Incident Cohort Kaplan-Meier graphs of the full cohort **(left)** and the incident treatment-naïve group **(right)**. Number at risk each year was presented below each plot. Log-rank test result comparing each group with Vol_high_Mass_low_ was shown, with Bonferroni p value. CMR = cardiac magnetic resonance.

Complete RV mass and volume data were available for 550 patients who were included in the group comparison study. We excluded 14 patients (2.5%) due to missing data. The VMI threshold was 0.53 (median). The numbers of patients in each group were as follows: 189 Vol_low_Mass_low_, 84 Vol_low_Mass_high_, 85 Vol_high_Mass_low_, and 192 Vol_high_Mass_high_. There were 69% of patients who had concordant remodeling (Vol_low_Mass_low_ and Vol_high_Mass_high_) and 31% had discordant remodeling (Vol_low_Mass_high_ and Vol_high_Mass_low_). Vol_low_Mass_low_ (HR: 0.390; 95% CI: 0.275 to 0.554; p < 0.001), Vol_low_Mass_high_ (HR: 0.260; 95% CI: 0.164 to 0.411; p < 0.001) and Vol_high_Mass_high_ (HR: 0.524; 95% CI: 0.373 to 0.734; p < 0.001) had lower mortality than Vol_high_Mass_low_. One-way analysis of variance test showed that Vol_high_Mass_low_ patients had the highest mean age (67 ± 13 years). At multivariate Cox regression analysis, Vol_high_Mass_low_ group (p = 0.001) was independent of age, sex, World Health Organization functional class, and pulmonary vascular resistance. For the subgroup analysis of incident treatment-naive patients (n = 383), the threshold for VMI was 0.55. Figure 1 illustrates the survival of patients by volume/mass group in the full and incident cohort.

Regarding limitations, the study cohort consisted of patients referred to a single tertiary center and would benefit from prospective validation. Assessment of longitudinal changes in volume/mass classification would be of great interest. The study used the previous diagnostic threshold of pulmonary hypertension (mPAP ≥25 mm Hg and pulmonary vascular resistance >3 WU).

The study has identified that Vol_high_Mass_low_ is a maladaptive cardiac phenotype in PAH and may be important to recognize to guide optimal counselling and therapy. Because individuals with Vol_high_Mass_low_ were older than other groups, one factor may be that, in older patients with significant pulmonary vascular disease, the RV maladapts with insufficient adaptive hypertrophy to maintain RV function. Vol_high_Mass_low_ remained prognostic following adjustment for age, suggesting that age does not fully explain the poor outcome, and, at all ages, Vol_high_Mass_low_ should alert the physician of high risk of adverse outcome. In contrast, Vol_low_Mass_low_ patients had less RV remodeling and maintained better RV function. Vol_low_Mass_high_ and Vol_high_Mass_high_ had better prognoses potentially through adaptive concentric hypertrophy that increases the RV wall thickness (3).
